# Book Review: Psychology and the Conduct of Everyday Life

**DOI:** 10.3389/fpsyg.2021.720997

**Published:** 2021-08-06

**Authors:** Bailong Liu, Kaixian Li

**Affiliations:** ^1^School of Marxism, Xi'an University of Architecture and Technology, Xi'an, China; ^2^School of Marxism, Xi'an Jiaotong University, Xi'an, China

**Keywords:** conduct of everyday life, critical psychology, human behavior, social psychology, methodology

From the earliest days of its formation as an independent discipline, psychology has been concerned with human experience, activity and self-reflection, but the question of how to conduct one's life has not received much attention (Carolyn, 2014). This book is about the psychological research and the conduct of everyday life in contemporary society. It brings psychological research from the laboratory to the real world. With its focus on the question of how people beings as active sensuous subjects live their everyday lives, it explores the conduct of life as a basis for comprehending the dilemmas and contradictions we face in daily lives.

Structurally, the book encompasses three parts, organized thematically into 13 chapters. The first chapters collect the latest interdisciplinary empirical research on the importance of environment. In the first chapter, it highlights the power of the concept of the conduct of everyday life to reform the perspective of psychology. In Chapter 2, a sociological approach to the research of the conduct of everyday life is introduced. Society can therefore be understood through the everyday lives of the individuals performing these actions. Chapter 3 presents the importance of everyday living for psychology, including a discussion of the sociological approach, an analysis of why the concept has not so far been investigated within psychology, as well as reflections on how to study the conduct of life from the standpoint of the subjects. In Chapter 4, it focuses on the importance of a specific practice of everyday living: walking and the practice of exposure as an alternative model of education. In Chapter 5, it reports the approach to the conduct of everyday life in the context of critical psychology. The study of the conduct of life needs to include concepts such as habitus, performativity, and privilege that are grounded in critical theories of embodiment and not in a philosophy of consciousness. Chapter 6 illustrates and reflects on how to carry out empirical research on everyday living. Everyday living is often conceptualized in terms of the mundane or ordinary. Yet, for increasing numbers of people disruption and the extra-ordinary have become normative (Highmore, 2002). In Chapter 7, based on empirical research on children's conduct of life, it focuses on conflicts in everyday life and their relatedness to social, political, and structural conflicts. This chapter discusses challenges of how to conceptualize meanings of the children's social backgrounds as well as how to conceptualize their personal agency. The starting point of everyday living in Chapter 8 is Adorno's famous dictum “there is no right life in the wrong one (Adorno, 2005).” Recognizing this dilemma is the first step out of it (p. 164). However, to overcome such a restricted concept of human agency and subjectivity, it is necessary to become aware of the varied forms and ways in which we unwittingly support in our own thoughts and actions conditions that we want to overcome. This includes the need to recognize and resist the many pressures that lead us to ignore all contradictory information so as to keep up the semblance of being able to live our lives in the right way, in contrast to others (Holzkamp, 2013).

The middle part examines the interaction between daily life throughout the world and contemporary global phenomena such as the rise of the debt economy, the hegemony of the labor market, and the increased reliance on digital technology in educational settings. Chapter 9 examines everyday life in the shadow of the rise of the debt economy in the United States. The chapter concludes with a historical sketch of the anti-debt movement in the United States that developed in the wake of the 2008 financial crisis and that potentially can challenge contemporary conduct of everyday life in the debt economy. Chapter 10 discusses these issues through exploration how the restructuring of the world economy has affected reproductive work and gender relations, the role of technology in this process, and the initiatives taken by women in the world to construct more cooperative and equitable forms of reproduction. Chapter 11 investigates the contradictory significance of digital technology in students' learning. The digitization of higher education is radically transforming learning and teaching relations, including the content of learning and the students' conduct of life. Based on a refined concept of learning it shows how the digitalization of students' learning environment reconfigures the structures of participation and how it can catalyze but also freeze the fluidity of learning and teaching.

Finally, the last part of the book is focused on how social psychology can enhance our understanding of our lives, and provide the possibilities for collective work to solve social conflict. The penultimate chapter explores the relationship between theory and everyday understanding as a basic challenge in the study of the conduct of life (p. 226). The chapter introduces memory-work as a possible approach to deal with this challenge and presents its individual steps, theoretical foundations, and possibilities for an empirical inquiry into everyday living. In the closing chapter, the authors expand the methodological discussion of how to empirically explore everyday living by focusing on the interconnections of subjective and structural aspects of persons conducting their everyday life in and across social practices (p. 241). This chapter elucidates the possibilities for arranging participatory research collaboration that enables the development of knowledge about common problems and contradictory life conditions in their meanings to different persons.

This book provides a basic introduction to the psychological research of the conduct of everyday life in contemporary society. Throughout the book, working with the “conduct of everyday life” and refining this concept can support an understanding of psychological phenomena as they unfold in the reality of everyday living, and promote a fundamental renewal of psychological theory, methodology, and practice. The importance of the conduct of everyday life for psychology lies in its conceptual relevance in exploring and understanding the everyday activities of individual subjects to organize, integrate, and make sense of the multiplicity of social relations and contradictory demands in and across the different contexts in which they are engaged in their daily life (p. 1). In a sense, the above content of this book is so extensive that some fascinating details, such as reflecting on the new methodologies and research practices facilitating the empirical investigations of the everyday realities and problems in people's lives, cannot be specifically presented. In this context, there is a growing need for psychology to investigate and understand how people confront and experience local changes in relation to social systems, institutions, technologies, and daily life practices in the course of their everyday life. Psychological theory and research, in turn, thus have to relate their understanding of human sensuous activities and experiences to the social practices and structures in which people live and experience their problems.

## Author Contributions

BL and KL wrote the manuscript, with larger contributions by BL. KL then provided edits and suggestions for revision. Both authors contributed to the article and approved the submitted version.

## Conflict of Interest

The authors declare that the research was conducted in the absence of any commercial or financial relationships that could be construed as a potential conflict of interest.

## Publisher's Note

All claims expressed in this article are solely those of the authors and do not necessarily represent those of their affiliated organizations, or those of the publisher, the editors and the reviewers. Any product that may be evaluated in this article, or claim that may be made by its manufacturer, is not guaranteed or endorsed by the publisher.
